# Gas6 and Protein S Ligands Cooperate to Regulate MerTK Rhythmic Activity Required for Circadian Retinal Phagocytosis

**DOI:** 10.3390/ijms25126630

**Published:** 2024-06-16

**Authors:** Célia Parinot, Jonathan Chatagnon, Quentin Rieu, Solène Roux, Dorine Néel, Florian Hamieh, Emeline F. Nandrot

**Affiliations:** Sorbonne Université, INSERM, CNRS, Institut de la Vision, 17 rue Moreau, F-75012 Paris, France; celia.parinot@gmail.com (C.P.);

**Keywords:** retinal pigment epithelium, phagocytosis, Mer tyrosine kinase (MerTK), Gas6, Protein S, ligand binding sites, retinal pigment epithelium, site-directed mutagenesis

## Abstract

Among the myriad of existing tyrosine kinase receptors, the TAM family—abbreviated from Tyro3, Axl, and Mer tyrosine kinase (MerTK)—has been extensively studied with an outstanding contribution from the team of Prof. Greg Lemke. MerTK activity is implicated in a wide variety of functions involving the elimination of apoptotic cells and has recently been linked to cancers, auto-immune diseases, and atherosclerosis/stroke. In the retina, MerTK is required for the circadian phagocytosis of oxidized photoreceptor outer segments by the retinal-pigment epithelial cells, a function crucial for the long-term maintenance of vision. We previously showed that MerTK ligands carry the opposite role in vitro, with Gas6 inhibiting the internalization of photoreceptor outer segments while Protein S acts conversely. Using site-directed mutagenesis and ligand-stimulated phagocytosis assays on transfected cells, we presently demonstrate, for the first time, that Gas6 and Protein S recognize different amino acids on MerTK Ig-like domains. In addition, MerTK’s function in retinal-pigment epithelial cells is rhythmic and might thus rely on the respective stoichiometry of both ligands at different times of the day. Accordingly, we show that ligand bioavailability varies during the circadian cycle using RT-qPCR and immunoblots on retinal and retinal-pigment epithelial samples from control and beta5 integrin knockout mice where retinal phagocytosis is arrhythmic. Taken together, our results suggest that Gas6 and Protein S might both contribute to refine the acute regulation of MerTK in time for the daily phagocytic peak.

## 1. Introduction

In the retina, daily vision is initiated in the photosensitive photoreceptor outer segments (POSs) where light is absorbed and also induces oxidative damage. Photoreceptors replace permanently their POSs and shed daily their aged extremities. Separated POSs are readily and rhythmically eliminated through phagocytosis by cells from the adjacent retinal pigment epithelium (RPE) [1]. This circadian activity of RPE cells reaches its maximum two hours after light onset [2]. The completion of phagocytosis is key to photoreceptor function and survival as its absence causes rapid photoreceptor degeneration due to debris accumulation [3]. When phagocytosis is completely arrhythmic, cumulative processes can arise and lead to age-related vision loss [4].

Similar to the elimination of apoptotic cells (ACs) by macrophages, RPE phagocytosis is a very organized process that follows sequential steps. First, ACs and POSs are recognized by αvβ3 and αvβ5 integrin receptors, respectively [5,6], via Mgf-E8, a soluble ligand that targets surface-exposed phosphatidylserines (PtdSer) [7,8,9]. In the retina, the increased exposure of PtdSer and activity of the αvβ5 integrin–Mfg-E8 couple are responsible for the synchronized burst of POS phagocytosis after light onset [4,8,10]. After target tethering, intracellular signaling pathways lead to the activation of the Mer tyrosine kinase (MerTK) receptor [4,11], the absence of this receptor resulting in early-onset photoreceptor death due to the accumulation of shed POSs [12,13]. The deregulation of MERTK function linked to gene mutations or overexpression leads to various pathologies in humans, ranging from retinal dystrophies [14,15] to lupus-like autoimmune phenotypes [16], atherosclerosis [17], and cancers [18]. MerTK has also been shown to regulate the number of ACs and POSs linked to macrophage and RPE cell surfaces [19,20].

MerTK is one of the three receptors of the TAM family, named after the respective initial of each receptor: Tyro3, Axl, and MerTK [21]. TAM receptors share very close structural features and are implicated in both the general homeostasis of tissues—via the removal of numerous loads of ACs throughout the body—and the downregulation of inflammatory responses [22]. Axl and MerTK have been shown to contribute to the high levels of efferocytosis reached by macrophages [23]. However, at the functional level, specificities exist: in macrophages, MerTK functions as a tolerogenic receptor, while Axl is mobilized by proinflammatory molecules [24], and receptor expression can be tissue-specific like in the retina, where only MerTK and Tyro3 are expressed [25].

TAM receptors share two cognate ligands, the vitamin K-dependent Gas6 and Protein S [26,27]. While their molecular structures are very similar, they are used in distinct functions [28,29]. Importantly, Protein S is implicated in AC clearance by macrophages [30]. First linked to PtdSer, Protein S binds to TAM receptors and mediates their activation by inducing receptor dimerization and autophosphorylation [31]. Additionally, Gas6 has the capability to bind PtdSer and stimulate receptor activation and efferocytosis [32,33,34], but its precise role in apoptotic cell clearance is not clear. Protein S alone seems to be sufficient to elicit phagocytosis [35]. However, both Gas6 and Protein S are expressed in the retina [25] and have been shown to stimulate POS phagocytosis by RPE cells [36]. For a long time, doubts were lingering about the in vivo utility of MerTK ligands for retinal phagocytosis, with their absence in single mutant mice not leading to any ocular phenotype [25,36,37]. Then, the creation by the Lemke group of a mouse model inactivated for both Gas6 and Protein S that develops blindness similarly to MerTK-deficient rodents made it clear that both ligands are required in the retina [37].

Interestingly, in β5 integrin (*β5*^−/−^) and Mfg-E8 (*Mfg-E8*^−/−^) knockout mouse eyes, the peak of POS phagocytosis is lost but phagocytosis persists [4,8], thus suggesting that MerTK might be stimulated by Gas6 and/or Protein S directly. In contrast to circulating macrophages, RPE cells are in permanent contact with POSs and the timeframe of peak MerTK phosphorylation is very sharp; thus, MerTK activation has to be regulated very tightly to avoid excessive phagocytosis to occur. Our data showed that MerTK function might be controlled by the cleavage and release of soluble MerTK (sMerTK) in the extracellular matrix both in vitro and rhythmically in vivo [38]. Very recently, we verified that hypothesis using MerTK cleavage-resistant (MerTK^CR^) mice for which the cleavage site had been removed genetically and which displayed a defective binding of phagocytosis in vitro and a deregulated rhythm of phagocytosis in vivo [39]. Surprisingly, and in contrast to macrophages, increasing doses of Gas6 and Protein S have opposite effects on sMerTK release and POS phagocytosis [38]. While Gas6 appears to increase sMerTK release and block phagocytosis, Protein S has the converse effects. These data suggest that both ligands might bear different roles in the regulation of MerTK function in the retina. Therefore, we analyzed the mRNA and protein expression profiles of Gas6 and Protein S to assess their respective bioavailability along the light–dark cycle. We also asked whether both ligands share the same binding site/s on the MerTK extracellular domain or whether they had specific binding patterns using a site-directed mutagenesis approach.

## 2. Results

### 2.1. Diminished MerTK and Mfg-E8 Expression in β5^−/−^ Mice Devoid of the Phagocytic Peak

**Figure 1:**
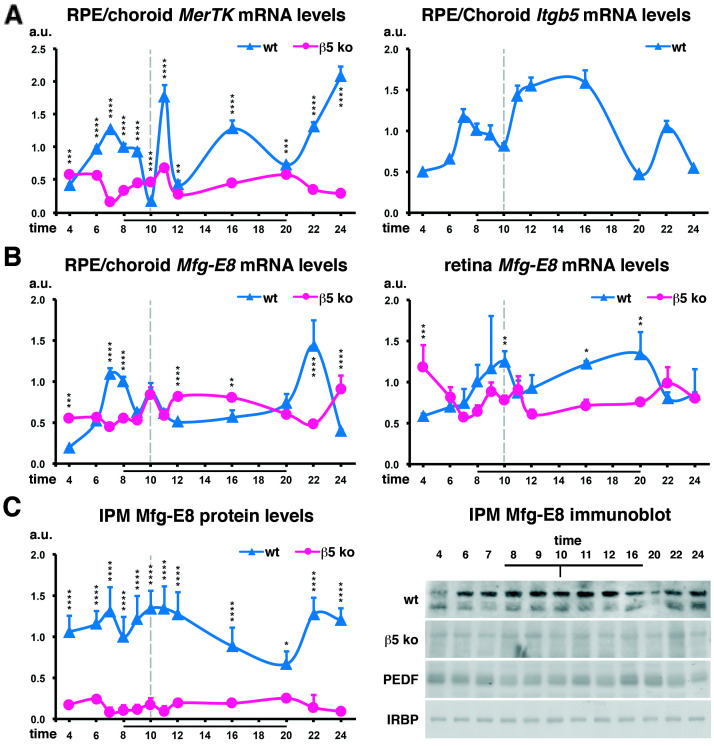
Rhythmicity of MerTK, Itgb5, and Mfg-E8 expression profiles along the light–dark cycle. Analysis of the mRNA (**A**,**B**) and protein (**C**) expression profiles for Mer tyrosine kinase (MerTK), β5 integrin (Itgb5, (**A**)), and Mfg-E8 (**B**) in the RPE/choroid, retina, or interphotoreceptor matrix (IPM) for wildtype (wt, blue) and *β5*^−/−^ mice (β5 ko, pink) at different times of day as indicated. (**A**) *MerTK* gene expression peaks before light onset, after peak phagocytosis, at 16.00 and 24.00 in wt animals while its expression is reduced and less rhythmic in *β5*^−/−^ mice. The *Itgb5* gene encoding the beta5 integrin protein follows a trimodal expression pattern, peaking at 7.00, 12.00–16.00, and 22.00. (**B**) In wt mice, *Mfg-E8* gene expression increases before light onset, at peak phagocytosis time, and at 22.00 in the RPE/choroid fraction, and before peak phagocytosis and in the afternoon up to light offset in the retina. In *β5*^−/−^ mice, only the increase at peak phagocytosis time in the RPE/choroid is maintained, but average expression levels do not change extensively. (**C**) Levels of Mfg-E8 ligands available in the IPM are increased at 7.00, around peak phagocytosis time, and at 22.00 as shown by quantifications and representative immunoblots. Levels of PEDF and IRPB controls do not vary. Results are shown in arbitrary units (a.u.) as means ± SDs, n = 3–9 independent samples; reference: wildtype sample at 8.00. * *p* < 0.05, ** *p* < 0.01, *** *p* < 0.001, and **** *p* < 0.0001; two-way ANOVA with a Sidak post-test comparing wildtype and *β5*^−/−^ samples at each time-point. Black bars: time-points during which lights were on (8.00–20.00); grey dotted bar: phagocytosis peak. A, *MerTK*, wt: modified from Law et al., The Journal of Biological Chemistry 2015 [38].

We showed previously that the expression of *MerTK* varies considerably during the light–dark cycle at crucial times, i.e., before the launch of phagocytosis at light onset and after the phagocytic peak just before and after it is required, as well as after light offset [38]. We investigated whether these expression levels were modified in *β5*^−/−^ mice whose phagocytosis was arrhythmic [4]. We observed both a great reduction in *MerTK* expression levels as well as the almost complete loss of *MerTK* rhythmic expression when phagocytosis was performed as a steady-state function (Figure 1A). Interestingly, the expression of *Itgb5*, the gene encoding the β5 integrin receptor, follows a similar pattern of expression as the one displayed by *MerTK*, with peaks before (before light onset) and after receptors are used (after peak phagocytosis). A third peak of expression is observed 2 h after light offset, when a potential second peak of phagocytosis might occur [40].

The αvβ5 integrin ligand Mfg-E8 is produced by both RPE cells and photoreceptors, and the expression of its gene also follows a rhythmic profile (Figure 1B). Its transcription in the RPE/choroid rises before light offset, at peak phagocytosis time, and 2 h after light offset. In the retina, its expression is increased just before the morning peak of phagocytosis and before light offset. At the protein level, the bioavailability of Mfg-E8 as a soluble ligand in the interphotoreceptor matrix (IPM) combines both profiles (Figure 1C). Interestingly, in absence of the phagocytic burst, very low quantities of ligands are present in the IPM when mRNA expression levels are only slightly reduced, suggesting a potential regulation at the post-transcriptional level.

### 2.2. Complementary Gas6 and Protein S Expression Profiles

**Figure 2:**
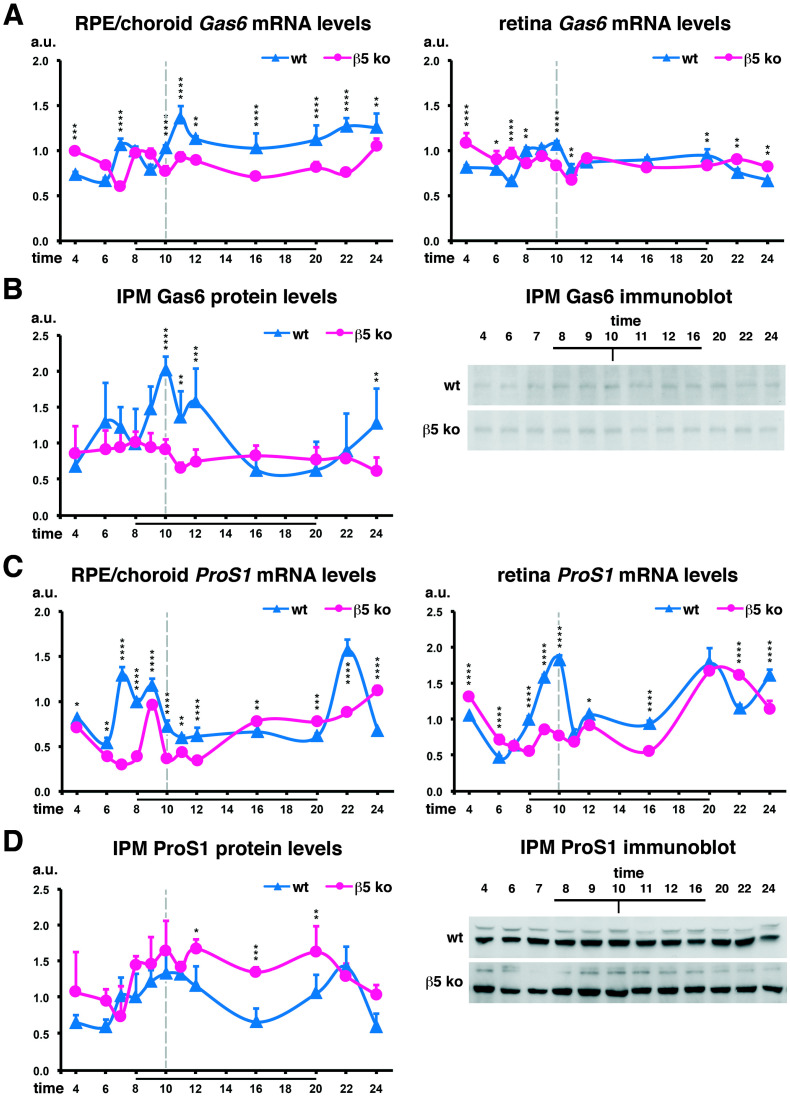
Gas6 and Protein S bioavailabilities peak at different times of the light–dark cycle. Analysis of the mRNA (**A**,**C**) and protein (**B**,**D**) expression profiles for Gas6 (**A**,**B**) and Protein S (**C**,**D**) in the RPE/choroid, retina, or IPM for wildtype (wt, blue) and *β5*^−/−^ mice (β5 ko, pink) at different times of day as indicated. (**A**) qPCR experiments allowed us to show that *Gas6* mRNA expression levels are slightly increased just before (retina) and after (RPE/choroid) the phagocytic peak in wt mice. *Gas6* expression levels were lower in the RPE/choroid of *β5*^−/−^ mice between peak phagocytosis time and 22.00 while levels were unchanged in the retina fraction. (**B**) Corresponding protein quantification and representative immunoblots in the IPM of fellow eyes showed a decrease at light onset followed by a marked increase at the time of the phagocytic peak in wt animals. In *β5*^−/−^ mice, expression levels did not vary. (**C**) *Pros1* mRNA expression increases just before and at the time of peak phagocytosis in wt RPE/choroid and retina, respectively. In both tissue samples, a second peak occurs at night offset (retina) of just after (RPE/choroid). The 7.00 and 22.00 RPE/choroid peaks, as well as the phagocytosis retina peak, are lost in *β5*^−/−^ mice, but median levels are not changed. (**D**) Corresponding protein quantification and representative immunoblots in the IPM of fellow eyes follows a combination of RPE/choroid and retina gene expression profiles depicted in (**C**). Results are in arbitrary units (a.u.) as means ± SDs, n = 3–8 independent samples; reference: wildtype sample at 8.00. * *p* < 0.05, ** *p* < 0.01, *** *p* < 0.001, and **** *p* < 0.0001; two-way ANOVA with a Sidak post-test comparing wildtype and *β5*^−/−^ samples at each time-point. Black bars: time-points during which lights were on (8.00–20.00); grey dotted bar, black tick: phagocytosis peak.

In wildtype animals, *Gas6* mRNA expression levels along the light–dark cycle slightly increase just before light onset, after the phagocytic peak, and remain elevated up to midnight in the RPE/choroid fraction while in the retina fraction, *Gas6* expression increases only before the phagocytic peak (Figure 2A). In contrast, *Gas6* mRNA expression profiles in *β5*^−/−^ mice are less rhythmic. In contrast, corresponding Gas6 protein levels in the IPM are markedly augmented at the time of maximum phagocytosis when MerTK needs to be deactivated for the phagocytic activity to stop (Figure 2B).

We could detect a bimodal profile of *Pros1* synthesis in both the RPE/choroid, at the time of light onset and after light offset, and in the retina, at the peak phagocytic time and at light offset in wildtype controls (Figure 2C). Amounts of Protein S available in the IPM are increased just before and at the time of the phagocytic peak, as well as at the putative evening peak (Figure 2D). Interestingly, in *β5*^−/−^ mice, part of the peak of *Pros1* expression before light onset (7.00–8.00) as well as the peak after light offset are lost, but ligand levels in the IPM are not really modified.

### 2.3. Importance of the Relative Expression Levels of Each Ligand

**Figure 3:**
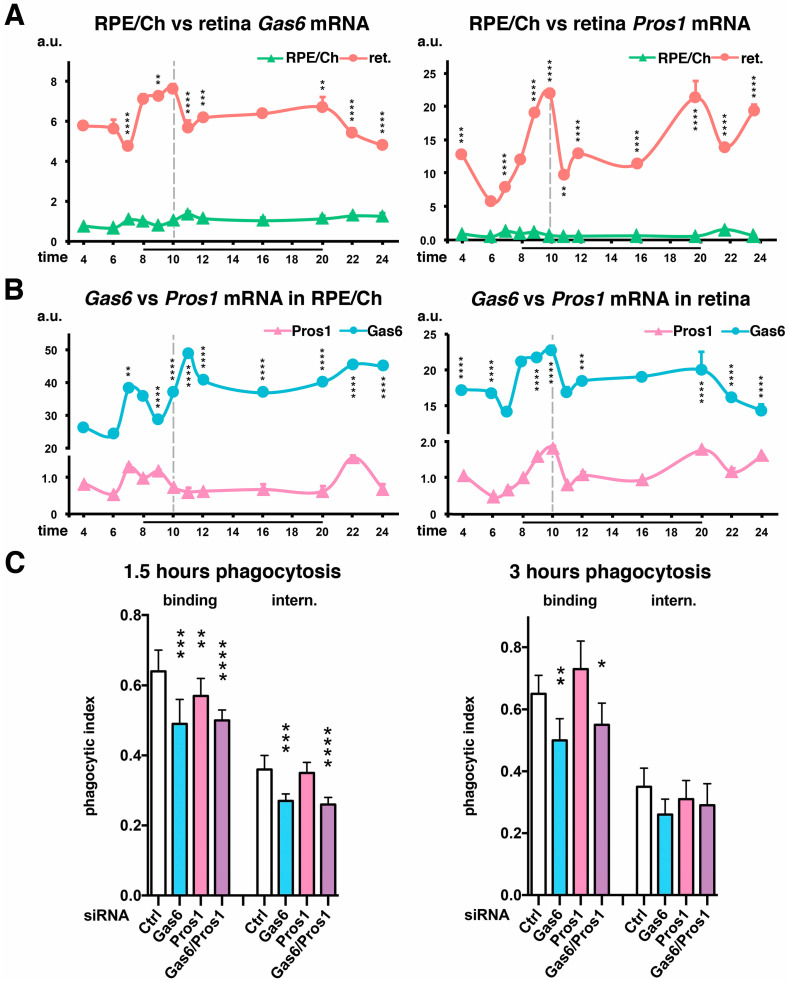
*Gas6* is more expressed than *Pros1*, and ligands are more expressed in the retina than in the RPE/choroid. (**A**) *Gas6* and *Protein S* (*Pros1*) mRNA expression profiles in RPE/choroid (green) and retina (orange) fractions of wildtype (wt) mice were compared at different times of day as indicated. Both ligands were more expressed in the retina than in the RPE/choroid. (**B**) Respective *Gas6* (blue) and *Pros1* (pink) mRNA expression profiles were compared in both the RPE/choroid and retina fractions of wt mice at different times of day as indicated. In both tissue types, *Gas6* was much more expressed than *Pros1*. (**A**,**B**) Results are in arbitrary units (a.u.) as means ± SDs, n = 3–7 independent samples; references: RPE/choroid (**A**) or *Pros1* (**B**) sample at 8.00. ** *p* < 0.01, *** *p* < 0.001, and **** *p* < 0.0001; two-way ANOVA with a Sidak post-test comparing wildtype and *β5*^−/−^ samples at each time-point. Black bars: time-points during which lights were on (8.00–20.00); grey dotted bar, black tick: phagocytosis peak. (**C**) siRNA samples were used to downregulate the endogenous production of each ligand by RPE-J cells. Cells were then subjected to phagocytosis assays for 1.5 and 3 h as indicated. Decrease in *Gas6* synthesis (blue bars) leads to diminished binding and internalization of POSs compared to control siRNA (Ctrl, white bars). Blocking the production of *Protein S* (*Pros1*, pink/purple bars) only slightly affects binding at 1.5 h. Adding both siRNAs has the same effect than adding the *Gas6* siRNA alone. Targeting of both ligands’ production (purple bars) has the same effect as the decrease in *Gas6* alone. Results of FITC/DAPI ratios are in arbitrary units (a.u.) expressed as means ± SDs, n = 5–6 independent experiments; * *p* < 0.05, ** *p* < 0.01, *** *p* < 0.001, and **** *p* < 0.0001, one-way ANOVA with a Tukey post-test compared to each series corresponding control; reference: total phagocytosis (binding + internalization) for the control condition.

If we compare gene expression levels between the RPE/choroid and retina fractions, both ligands have higher levels of mRNA produced in the retina, between five and eight times for *Gas6* and five and twenty-two times for *Pros1* (Figure 3A). In addition to their expression in photoreceptors, these elevated expressions in the retina might also be due to the high number of blood vessels spanning the inner retinal network that express these ligands consistently.

Our recent data suggest that, in contrast to macrophages, Gas6 and Protein S assume converse roles in POS phagocytosis [38]. Increasing doses of Gas6 inhibit POS internalization while doses of Protein S stimulate POS engulfment. Earlier studies including by the Lemke group showed that both ligands are interchangeable for phagocytosis completion, and their joint absence leads to a retinal degeneration phenotype similar to the one displayed when MerTK is not functioning or absent [36,37]. Therefore, the presence of respective quantities of each ligand in the IPM along the light–dark cycle is important in order to control this rhythmic activity. At the gene level, *Gas6* is substantially more expressed than *Pros1* at any given time in both RPE/choroid (×24–48) and retinal (×14–23) samples (Figure 3B).

With ligands being produced by both photoreceptors and RPE cells, we tested the effect of blocking the RPE endogenous expression of each ligand separately or at the same time on the phagocytic abilities of rat RPE-J cells via siRNA inhibition and without the addition of exogenous ligands to the medium. Decreasing *Gas6* expression directly impacted, significantly both steps of phagocytosis, binding after 1.5 and 3 h of POS challenge and internalization after 1.5 h, while *Pros1* downregulation only impacted binding after 1.5 h of phagocytosis (Figure 3C). No additional effect was gained by blocking the expression of both ligands compared to blocking Gas6 alone. We noticed that the effects are more marked early on at 1.5 h than after 3 h of phagocytosis, suggesting that in vitro ligand implication is mostly used in the early steps of phagocytosis and less for the subsequent internalization when MerTK is activated by intracellular pathways initiated by αvβ5 integrin receptors [4,41].

### 2.4. Gas6 and Protein S Recognize Different Amino Acids of MerTK Ig-like Domains

**Figure 4:**
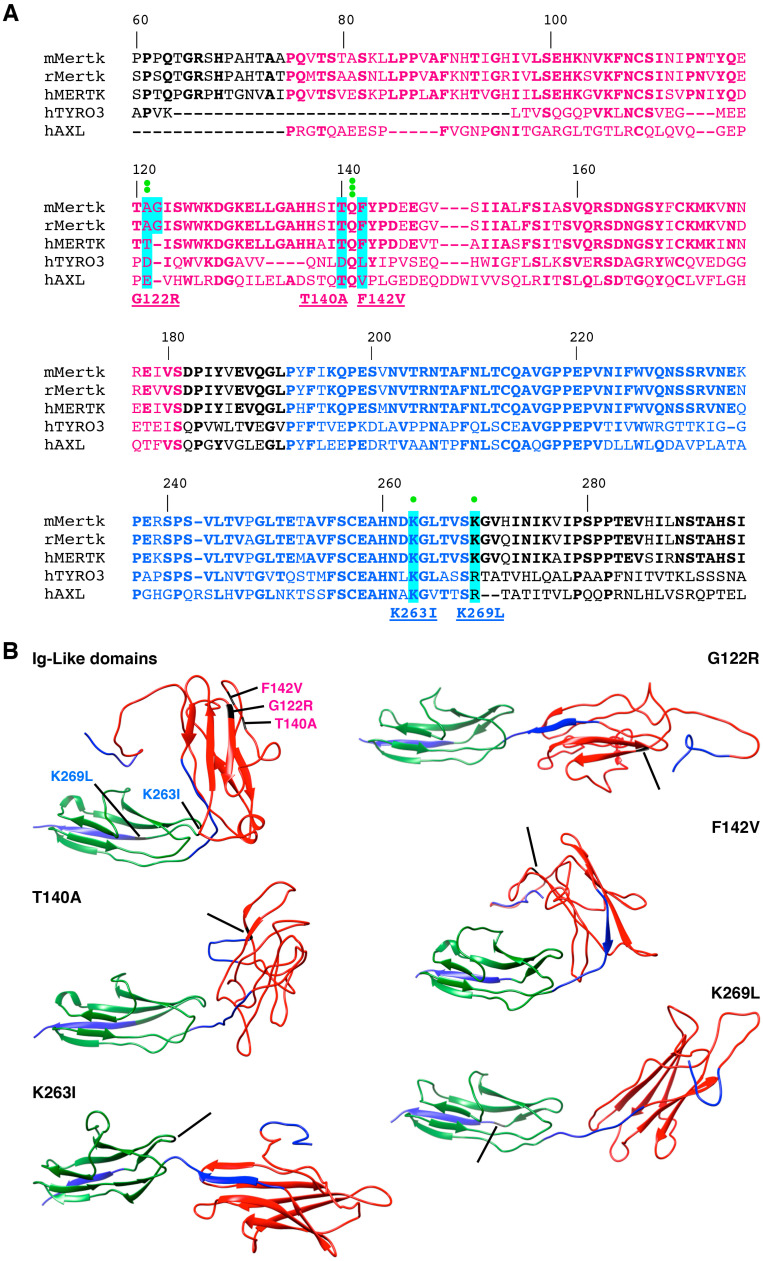
TAM sequence alignments and 3-D structures of MerTK ligand binding site mutants. (**A**) Sequence alignment of Ig-like 1 (pink) and 2 (blue) domains for mouse (m), rat (r), and human (h) MerTK; human Tyro3; and human Axl. Identical amino acids are shown in bold and mutated amino acids are highlighted in blue, named, and underlined. Green dots show critical areas identified previously on Axl [41]. (**B**) Three-dimensional structure of Ig-like domains 1 (red) and 2 (green) in control (Ig-like domains) and 5 individual mutants as indicated. The Phyre2 web portal was used for mouse MerTK (NM_008587) ligand-binding-site modeling and structure prediction (http://www.sbg.bio.ic.ac.uk/phyre2/html/page.cgi?id=index, accessed on 4 August 2017) [43]. Molecular graphics and analyses were performed with the UCSF Chimera package developed by the Resource for Biocomputing, Visualization, and Informatics at the University of California, San Francisco (https://www.cgl.ucsf.edu/chimera/docs/credits.html, accessed on 9 August 2017) [44]. Black bars point out modified amino acids, the blue domain corresponds to the region between Ig-like domains 1 and 2.

In the TAM family, only the binding between Gas6 and Axl has been studied [42]. Using the crystallography analysis of ligand–receptor interaction and mutagenesis, researchers have identified a minor and a major binding site on Axl and shown that Gas6 binding to an Axl monomer elicits the dimerization and activation of the receptor. We decided to explore these two sites on the MerTK receptor regarding the binding of Gas6 and Protein S. For each site, located on each of the two Ig-like domains, we chose two to three relevant amino acids to target, referring to residues important to Axl [42] and sequence homologies between human Axl, human Tyro3, and mouse, rat, and human MerTK (Figure 4A). Amino acids were changed so that new residues displayed similar size and chemical properties (Table 1). We used the Phyre2 web portal to predict the 3-D structure of the Ig-like domains for each mutant, and illustrations were generated using the UCSF Chimera package [43,44] (Figure 4B).

**Figure 5:**
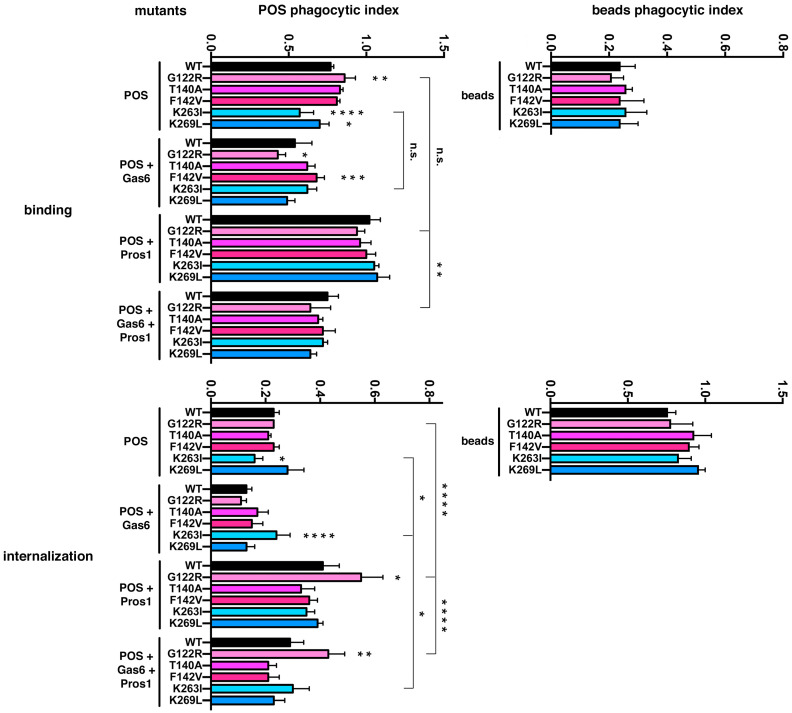
Gas6 and Protein S bind to different amino acids of MerTK Ig-like domains. Mutants targeting ligand binding sites in MerTK Ig-like domains 1 (pink bars) and 2 (blue bars) were transfected in RPE-J and tested for their influence on POS binding (top left) and internalization (bottom left) when compared to non-mutated MerTK (black bar) with or without the addition of Gas6 and Protein S—alone or in combination—as indicated. The p.Gly122Arg (G122R, light pink bars) mutant significantly increases POS binding in DMEM while addition of Gas6 diminishes binding and addition of Protein S importantly increases internalization compared to the wt construct. Among the neighbor sites p.Thr140Ala (T140A) and p.Phe142Val (F142V), only p.Phe142Val (F142V) shows a slight increase in POS binding in the presence of Gas6. The p.Lys263Ile (K263I) mutant has a negative impact on both binding and internalization of POSs alone, as well as a positive effect on the internalization of POSs with Gas6. The p.Lys269Leu (K269L) mutant has almost no effect besides slightly less binding of POSs alone. When challenged with fluorescent beads (right bar graphs), no difference was observed in this study between the different clones. Results of FITC/DAPI ratios in arbitrary units (a.u.) are expressed as means ± SDs, with n = 4–6 independent experiments (POSs, left) or n = 3–4 independent experiments (beads, right). * *p* < 0.05, ** *p* < 0.01, *** *p* < 0.001, and **** *p* < 0.0001; one-way ANOVA with a Tukey post-test compared to each series corresponding wildtype; reference: total phagocytosis (binding + internalization) for the control condition (WT). Significance brackets compare different ligand conditions for a single mutant.

RPE-J cells, expressing endogenous MerTK, were transfected with the different clones. The normal *MerTK* cDNA reacted to ligand stimulation as shown previously on native RPE-J: Gas6 inhibited phagocytosis while Protein S enhanced it (Figure 5) [38]. The addition of each ligand separately or together allows us to test the binding affinity of each mutant. In the Ig-like 1 domain, the biggest effect was observed with the G122R amino acid change that lowered the binding of POSs with Gas6 by 20 ± 2% (*p* < 0.05) and enhanced, greatly, their internalization with Protein S alone by 35 ± 5% (*p* < 0.05) or with Gas6 and Protein S by 47 ± 7% (*p* < 0.01). The addition of Gas6 seems to slightly increase POS binding by the F142V mutant (+26 ± 2%, *p* < 0.001). The second most impactful amino acid change was of K263I in the Ig-like 2 domain: it impeded both the binding and internalization of POSs alone (−26 ± 4%, *p* < 0.0001, and −30 ± 5%, *p* < 0.05, respectively) but strongly ameliorated POS internalization in the presence of Gas6 (+84 ± 18%, *p* < 0.0001). In contrast, the other mutant, K269L, only slightly affected the binding of POSs alone (−9 ± 1%, *p* < 0.05). Interestingly, similar phagocytosis assays using inert 1 µm fluorescent beads did not show any difference in the phagocytic capabilities of the various clones tested (Figure 5).

## 3. Discussion

Retinal phagocytosis and the clearance of ACs by macrophages share common features, which allows us to make analogies: the sequential organization of the process, similar molecular machinery, and cross-recognition of each other’s targets [45]. However, in the retina, the situation is considerably more complex due to the permanent contact between POSs and RPE cells and to the circadian regulation of phagocytosis. This characteristic makes it necessary for RPE cells to have a perfect control of the numbers of POSs they engulf to avoid achieving too much phagocytosis and damaging the homeostasis of the retina. Conversely, RPE cells need to properly eliminate damaged POSs in order to maintain photoreceptor health and vision [3]. Therefore, RPE phagocytosis functions as a sharp burst of activity that occurs once a day. At light onset, a series of events launched by αvβ5 integrin receptors are taking place, leading to the stimulation of MerTK, whose phosphorylation profile matches the peak of POS engulfment [4]. MerTK gene expression is also rhythmic, with a burst of synthesis just after phagocytosis and after light offset. We now show that in *β5*^−/−^ mouse RPE cells without the rhythmic clearance of POSs, the cyclic profile of MerTK expression is lost. Additionally, there is a great decrease in Mfg-E8 ligand quantities present in the IPM. This suggests that the rhythmicity of POS elimination and/or expression of *Itgb5* are required for rhythmic *MerTK* transcription and Mfg-E8 secretion.

Aside from its extracellular activation via αvβ5 integrin receptors, there are other ways by which MerTK activity can be controlled, including the stimulation by its two ligands. Interestingly, in contrast to macrophages, Gas6 appears to bear an inhibitory role while Protein S acts more as a stimulator of RPE cell phagocytosis [38]. Hence, the respective stoichiometry of these ligands might be crucial to control the balance of MerTK activity. Therefore an important question arises: when are MerTK ligands expressed and bioavailable for RPE cells? For the first time, we extensively analyzed the circadian expression of these ligands at the gene and protein levels in vivo. Protein S quantities increase from 1 h before light onset and decrease just after the phagocytic peak. On the other hand, the availability of Gas6 ligands in the IPM increases at the peak phagocytic time, when MerTK function must be downregulated for the peak to fade. Interestingly, in *β5*^−/−^ mice that display an arrhythmic phagocytosis, Gas6 ligands are present in the IPM in steady-state quantities. This suggests that in vivo, Gas6 might be the ligand negatively controlling MerTK’s activation state. However, components regulating its expression/secretion and that might be directly linked to the phagocytic function still need to be identified. As of note, the amplitude of the phagocytic peak is more marked in mice with the 129T2/SvEmsJ genetic background (*β5*^−/−^ mice) [4] than in C57BL/6J mice [39,46]. Hence, it is possible that the variations in gene expression we observed in this study might be attenuated in other mouse models on the C57BL/6J background. Additionally, it would be interesting to explore these gene expression patterns in other mouse models that display deregulated or arrhythmic POS phagocytosis without developing any phenotype [47,48]. In addition, both ligands are synthetized by both RPE and photoreceptors, and their expression profiles in the IPM appear to be combinations of both origins. Therefore, we still cannot exclude that both cell types contribute to the regulation of MerTK activity and thus phagocytosis. Interestingly, in human eyes, *GAS6* is much more expressed in the macular region that concentrates cone photoreceptors [49]. It would hence be interesting to assess whether this MerTK ligand plays a more important role in cones than in rods in humans.

Interestingly, the *Gas6* gene is, on average, expressed between 15% (retina) and 40% (RPE/choroid) more than *Pros1*. Unfortunately, we could not identify any kit accurate enough to quantify Protein S concentrations in our mouse samples in order to quantify the respective concentrations of each ligand in the IPM. While their retinal half-lives are not known, it is recognized that the affinity of MerTK for Gas6 is higher than for Protein S, and this might play an important role in the retina when compared to other tissues for which the contact between the phagocyte and its target is not permanent, suggesting that both ligands might contribute together to MerTK activity [35,50,51]. Therefore, the marked increase in Gas6 availability from the peak phagocytosis time might change the stoichiometry between the two ligands. In cancer cells, differential Gas6/Protein S ratios also exist, and higher ratios are directly associated with aggressiveness, showing the importance of respective quantities of each ligand [52]. Additionally, the change in Gas6/Protein S ratios could also modify the 3-D structure of the receptor and thus its dimerization/activity. In addition, some other factors in the IPM might participate in the process, such as calcium ions and ligand gamma-carboxylation, both required for the full activation of TAM receptors [33,35].

So far, the exact binding site/s for Gas6 and Protein S on MerTK has/have never been explored in any tissue. Here, using mutagenesis and phagocytosis assay approaches, we have shown that for POS phagocytosis, both Ig-like domains seem to be mobilized, most probably in relation with their 3-D structure generating a ligand pocket between MerTK dimers [42]. While Gas6 seems to be linked to both Ig-like domains, Protein S appears to recognize solely the Ig-like 1 domain. Thus, Gas6 and Protein S may act on different amino acids, raising the idea of a potential competition between the two ligands that could underlie the functional variations of MerTK activity. The same opposite functional effect of these two ligands seems to exist as well for Axl receptors, with Gas6 promoting survival and proliferation while Protein S leads to apoptosis [52]. Interestingly, a previous study of Axl binding demonstrated that for this receptor, the Ig-like 1 domain bears a higher affinity for Gas6 than the Ig-like 2 domain [42]. In addition, the binding of Gas6 to Axl uses amino acids in the ligand structure that are absent in Protein S, which might explain why Axl is not able to bind Protein S while MerTK and Tyro 3 can bind both ligands [42,51]. If we extrapolate this aspect to other TAM receptors, it confirms that ligand and receptor specificity and affinity can be based on slight differences in their respective sequences.

Thus, the results of this study reinforce and complement our previous work that suggested an opposite effect of Gas6 and Protein S on MerTK function in RPE cells, linked to the Gas6-related release of soluble MerTK receptors acting as decoys in the IPM [38]. When combining our in vitro and our in vivo data, we can make some hypothesis on the role of MerTK ligands in the fine regulation of the receptor’s activity. In RPE cells, Protein S appears to help stimulate MerTK in time for phagocytosis to reach its maximum and decreases thereafter. In contrast, Gas6 seems to sustain its inhibitory role through its increased bioavailability when phagocytosis has reached its peak and needs to be downregulated. Therefore, both ligands appear to contribute directly to the acute regulation of the phagocytic rhythm in the retina, aside from their requirement for MerTK dimerization [38]. Taken together, in this tissue where finely regulating MerTK activity is crucial, the differential participation of MerTK ligands in phagocytosis modulation might be ensured by the use of separate fixation sites on MerTK Ig-like domains, potentially influencing MerTK cleavage as suggested by our previous paper [38].

Overall, progress on the deciphering of the role of MerTK receptors and their ligands Gas6 and Protein S in phagocytic cells, as well as their contribution in the control of inflammation in multiple tissues, has been tremendous in over two decades, thanks to the founding research by Prof. Lemke’s laboratory and teams created by previous members from his team as well as to other contributors focusing on specific aspects or tissues such as the eye. Recent studies have been changing some paradigms, and there are still questions to be answered, especially for the understanding of the specific context of the retina where phagocytosis has to be tightly controlled due to the permanent contact between phagocytic RPE and apoptotic photoreceptor extremities.

## 4. Materials and Methods

### 4.1. Reagents and Antibodies

Reagents were from Life Technologies SAS (Courtaboeuf Cédex, France), unless otherwise stated. Gas6 recombinant proteins (mouse, 986-GS), as well as anti-mouse antibodies raised in goats against Gas6 (AF986) and MFG-E8 (AF2805), were from R&D Systems (Bio-Techne, Noyal-Châtillon-sur-Seiche, France). Recombinant human Protein S was from MP Biomedicals (194081) (Eschwege, Germany). Other antibodies were directed against IRBP (sc-18598, Santa Cruz Biotechnology, Inc.) (Dallas, TX, USA), PEDF (MAB1059, Millipore) (Sigma-Aldrich Chimie S.a.r.l., Saint Quentin Fallavier, France), and Protein S (ab97387, Abcam) (Amsterdam, Netherlands).

### 4.2. Animals

Homozygous β5 integrin knockout mice (*β5*^−/−^, RRID:IMSR_JAX:004166) [53] and wildtype (wt) mice from the same genetic background (129T2/SvEmsJ; The Jackson Laboratory) were housed under cyclic 12 h light:12 h dark conditions (light onset at 8.00 AM) and fed ad libitum. Animals were handled according to the Association for Research in Vision and Ophthalmology (ARVO) Statement for the Use of Animals in Ophthalmic and Vision Research. Protocols were approved by the Charles Darwin Animal Experimentation Ethics Committee from the Sorbonne Université and the French Ministry for Higher Education and Research under APAFIS #1631-2015090415466433 v3 and #20191-2019040311402311 v6. For experiments, male and female wt and *β5*^−/−^ mice aged from 2 to 4 months were euthanized through CO_2_ asphyxiation. Mice were sacrificed at 12 different time-points along the light–dark cycle as follows: 4.00, 6.00, 7.00, 8.00 (light onset), 9.00, 10.00 (phagocytosis peak), 11.00, 12.00, 16.00, 20.00 (light offset), 22.00, and 24.00. Eyes were gently removed and dissected in HBSS without CaCl_2_ and MgCl_2_. After removal of the lens, the retina was carefully separated from the rest of the cup—containing the RPE/choroid—in a dry dish and both parts were frozen separately in liquid nitrogen. One eye (cup and retina) from each animal was used for gene expression level assessment and the fellow eye for protein level analysis (see respective sections below).

### 4.3. RNA Extraction, Reverse Transcription, and Real-Time Quantitative PC

Individual sample RNAs were extracted using the Illustra RNAspin Mini RNA Isolation Kit (GE Healthcare, Cytiva, Saint-Germain-en-Laye, France) according to the manufacturer’s instructions with a second DNAse step to reduce the potential residual content of genomic DNA as previously described [38]. RNA yield and purity were measured using the NanoDrop ND-2000 spectrophotometer (Thermo Fisher Scientific, Illkirch, France). A quantity of 250 ng of total RNAs from each sample was loaded on a 1% agarose/1X TAE gel to check the RNAs’ integrity. A quantity of 500 ng of total RNAs from both retina and RPE/choroid of each eye was reverse-transcribed in a 50 μL final reaction volume for 1 h at 42 °C following the manufacturer’s protocol (Reverse Transcription System, Promega, Charbonnières, France).

PCR primers designed by us allowed the amplification of 150 bp fragments for all tested genes and the ribosomal protein Rho0 (*Rplp0*) housekeeping control (Table 2). Triplicate qPCR reactions for each sample were carried out on a 7500 Fast Real-Time PCR System using the Power SYBR Green PCR Master Mix (both from Applied Biosystems, Waltham, MA, USA) as follows: 50 °C for 2 min and 95 °C for 10 min, followed by 40 cycles of 95 °C for 15 s and 60 °C for 1 min. Reaction products were tested on 1.2% agarose/1× TAE gel electrophoresis and melting curves were analyzed to confirm size and specificity, respectively. Relative quantities of each gene were calculated using the 2^−ΔΔCt^ method and expression levels at 8.00 (light onset) were set as 1 as specified in each figure legend.

### 4.4. Retrieval of Soluble Proteins, Sample Lysis, and Immunoblotting

Soluble proteins from the IPM of mouse eyes were isolated from each sample in HBSS without CaCl_2_ and MgCl_2_ for 20 min on a shaker at 4 °C as described previously [38]. Samples were centrifuged at maximum speed for 5 min at 4 °C, then supernatants were ultra-centrifuged at 110,000× *g* for 30 min at 4 °C (Sorvall M120 SE Discovery, S120-AT2 rotor).

Tissue pellets were solubilized in 1% Triton X-100, 1% sodium deoxycholate, 0.1% SDS, 50 mM HEPES, 150 mM NaCl, 10% glycerol, 1.5 mM MgCl_2_, and 1 mM EGTA with 1% each of protease and phosphatase inhibitor cocktails. Isolated samples representing 20% of soluble proteins from the IPM mixed at 1:1 ratio between separated retina and RPE/choroid fractions from the same sample and whole-cell lysates representing approximately 7.5% of one tissue sample were separated on SDS–polyacrylamide gels and electroblotted onto nitrocellulose membrane (Protran 0.2 µm, Whatman, Maidstone, UK). Immunoblots were blocked with 10% milk in 1× TBS for 2 h, then probed with primary antibodies overnight and secondary antibodies for 2 h at RT. Signals were detected using a chemiluminescence detection system (Western Lightning Plus-ECL, PerkinElmer, Revvity, Villebon sur Yvette, France) and chemiluminescence films (Hyperfilm ECL, Amersham, Cytiva, Saint-Germain-en-Laye, France). Non-saturated films were scanned and signals quantified using NIH ImageJ 1.43u. Duplicates of an identical control sample were loaded on each immunoblot and used as references for protein quantifications and comparisons between different sample series.

### 4.5. MerTK cDNA Mutagenesis

Mouse MerTK cDNA with an extracellular GFP tag cloned into the OmicsLink Expression Clone vector (EX-Mm03855-M29) was purchased from GeneCopoeia (Rockville, MD, USA). Point mutations on the minor and major ligand binding sites were designed according to critical amino acids for ligand binding identified on Axl in a previous study [42] and using sequence alignments between Axl, Tyro3, and MerTK from different species (Figure 4A). Primer sequences were generated using the QuickChange Primer Design Program (www.agilent.com/genomics/qcpd, last accessed on 11 February 2019; Agilent Technologies, Santa Clara, CA, USA) (Table 1). Desired changes were obtained using the QuickChangeII XL Site-Directed Mutagenesis Kit (Agilent Technologies) with the following PCR amplification conditions: 5 min at 95 °C; 18 cycles of 1 min at 95 °C, 1 min at 60 °C, and 22 min at 68 °C; and 7 min at 68 °C. Selected clones were confirmed via direct sequencing and were further amplified to generate plasmid stocks.

### 4.6. Cell Culture and Transfection

The rat RPE-J cell line (ATCC) was maintained at 32 °C and 5% CO_2_ in DMEM with 4% CELLect Gold FCS (MP Biomedicals, Eschwege, Germany), supplemented with 10 mM HEPES and 1% non-essential amino acids. For experiments, RPE cells were plated into alcian blue-coated 96-well plates. After 24 h, cells were transfected with the different expression plasmids for 5 h using Lipofectamine 2000 (Life Technologies SAS, Courtaboeuf Cédex, France) according to the manufacturer’s protocols, then used 72 h later. Alternatively, cells were transfected with rat ON-TARGETplus SMARTpool siRNAs (Gas6 L-088897-01, Pros1 L-097256-01) for 4 h using the DharmaFECT 4 siRNA Transfection Reagent as instructed (all from Dharmacon, Horizon Discovery, Cambridge, United Kingdom). Transfection efficiency was assessed using the siGLO RISC-free Control siRNA (D-001600-01) and specificity of the effects observed was validated through comparison with the ON-TARGETplus Non-targeting Pool (D-001810-10).

### 4.7. POS Isolation and Phagocytosis

POSs were isolated from porcine eyes fresh from the slaughterhouse according to an extensively described protocol [54]. Cells were challenged with approximately 10 POSs per RPE cell resuspended in DMEM. Alternatively, phagocytosis assays were performed using 1 µm polystyrene FluoSpheres (Life Technologies). In some assays, recombinant MerTK ligand proteins at 10 μg/mL were added to the POS suspension before challenging the cells for 3 h at 37 °C as previously described [38]. Cells were then washed three times with PBS-CM (0.2 mM Ca^2+^ and 1 mM Mg^2+^) at the end of the incubation. For each different condition, an incubation of half of the wells with trypan blue (Gibco, Life Technologies SAS, Courtaboeuf Cédex, France) for 10 min quenched the fluorescence of surface-bound FITC-labeled POSs, thus allowing us to quantify only POS internalization and calculate surface binding [6], followed by two more PBS-CM washes. After a fixation step of 10 min with ice-cold methanol for all wells, nuclei were counterstained with DAPI (Interchim). FITC-POS- and DAPI-labeled nuclei-derived signals were quantified using a microplate fluorescence reader (Infinite M1000, Magellan 6 software, Tecan, Männedorf, Switzerland).

### 4.8. Statistical Analysis

Depending on the assay, experiments were repeated on 5 to 9 independent samples as indicated in figure legends, except for bead phagocytosis assays (n = 3–4). Obvious outliers were removed from the calculations on the basis of the highest difference from the calculated mean and only if the deviation amounted to twice the maximum deviation observed without this particular value, all series being treated equally to avoid any bias. Significance of results was assessed using one-way ANOVA with a Tukey post-test or two-way ANOVA with a Sidak post-test as detailed in each figure legend. Significance levels are depicted here as follows: * *p* < 0.05, ** *p* < 0.01, *** *p* < 0.001, and **** *p* < 0.0001.

## Figures and Tables

**Table 1:** List of mutants chosen on the 2 Ig-like domains containing the putative ligand binding sites of mouse MerTK extracellular domain (NM_008587) and associated amino acid and nucleotidic changes.

Domain	Amino Acid Change	Sequence Change
**Ig-like 1**	p.Gly122Arg	c.364G>C
	p.Thr140Ala	c.418A>G
	p.Phe142Val	c.424T>G
**Ig-like 2**	p.Lys263Ile	c.788A>T
	p.Lys269Leu	c.805A>C, c.806A>T

**Table 2:** Gene names, accession numbers and corresponding sequences of oligonucleotides used for quantification of gene expression via qPCR (150 bp fragments).

Mouse Gene Accession #	Forward Primer	Reverse Primer
*Rplp0* NM_007475.5	CCTGAAGTGCTCGACATCAC	TGCCAGGACGCGCTTGTAC
*MerTK* NM_008587	CGTCTGTCCTAACCGTACCT	GTACTGTTGAGGATATGGACT
*Itgb5* NM_001145884	GGTTTCGGGTCTTTTGTTGAC	ACTCTGTCTGTGAGAGGCAG
*Mfg-E8* NM_008594.2	GCCTGAAGGTTAACATGTTCA	GTGTTATTCTTCAGGCCCAG
*Gas6* NM_019521.2	ATCAACCACGGCATGTGGC	CGGTGAGATTCAGGTGATAG
*Pros1* NM_011173.2	GCAGGAGTTGTCTTATATCTG	CACGAAGCGCAATCAGGAG

## Data Availability

Data is contained within the article, complete raw data can be provided upon request.
